# Nanoparticles of conformation-stabilized canine distemper virus hemagglutinin are highly immunogenic and induce robust immunity

**DOI:** 10.1186/s12985-021-01702-0

**Published:** 2021-11-22

**Authors:** Jingjian Dong, Yan Chen, Lili Shi, Bing Shen, Xianliang Sun, Kaiyi Ruan, Xianzhu Xia, Hao Feng, Na Feng

**Affiliations:** 1 Medical School of Jiaxing University, Jiahang Road 118#, Nanhu District, Jiaxing City, Zhejiang Province 314001 People’s Republic of China; 2 Military Veterinary Research Institute of Academy of Military Medical Sciences, Changchun, 130122 People’s Republic of China

**Keywords:** Canine distemper virus, Nanoparticle, Mucosal immune response, Hemagglutinin (H) tetramer, Giant panda

## Abstract

**Background:**

Canine distemper virus (CDV) infection of ferrets, dogs, and giant pandas causes an acute systemic disease involving multiple organ systems, including the respiratory tract, lymphoid system, and central nervous system. In this study, we tested a new candidate CDV vaccine-CDV nanoparticles-based on hemagglutinin protein.

**Methods:**

The nanoparticles were generated from conformation-stabilized CDV hemagglutinin tetramers. Immune responses against CDV were evaluated in mice. Immunization was initiated 6 weeks after birth and boosted two times with 4-week intervals. The blood and mucosal samples were collected 2 weeks after each immunization.

**Results:**

Vaccination with CDV nanoparticles elicited high levels of IgG antibody titers in mice (approximately sevenfold to eightfold higher than that obtained with soluble CDV H protein) and mucosal immune responses and developed increased CDV-specific neutralizing antibody. The mice that received nanoparticles showed significantly higher IFN-γ- and IL-4-secreting cell population in the spleen and lymph node compared with mice immunized with soluble H protein. The co-stimulatory molecular expression of CD80 and CD86 on the surface of DCs was also upregulated.

**Conclusion:**

The results demonstrate that self-assembly into nanoparticles can increase the immunogenicity of vaccine antigens, and nanoparticles assembled from conformation-stabilized CDV H protein can serve as a new CDV vaccine.

## Introduction

Canine distemper virus (CDV) is an enveloped, single-stranded RNA virus commonly found in the genus *Morbillivirus* and family *Paramyxoviridae*, which is closely related to measles virus (MV) [1]. The virus enters the hosts through the respiratory tract and targets immune cells, and after amplification in lymphoid organs, it disseminates via the blood stream to multiple organs, leading to gastrointestinal, dermatological, and respiratory signs [2, 3]. Canine distemper (CD), which is caused by CDV, is a highly contagious and fatal disease in a wide range of mammals [4, 5]. CDV infections have been observed in the order Carnivora, nonhuman primates, rhesus monkeys, and giant pandas [6–8]. Four pandas infected with CDV died in Chongqing Zoo and Nanjing Zoo [9]. Six giant pandas with confirmed CDV infection in the Shanxi Rare Wild Animal Rescue and Research Center in China were isolated and labeled as SX/2014 [8].

Current CDV vaccines include subunit vaccine, attenuated vaccine, inactivated vaccine, and DNA vaccine, and many kinds of CDV vaccine approaches have been evaluated at present. Previous studies on CDV have utilized purified hemagglutinin (H) and fusion (F) proteins to immunize small numbers of dogs against CDV challenge [10–12]. In addition, the DNA vaccine that expresses the CDV H protein is sufficient to protect minks against *Morbillivirus* infection [13], and other vector vaccines also show superior immunogenicity compared with live, attenuated vaccine [14]. Inactivated or killed vaccine tends to stimulate a weak immune reaction and requires the administration of multiple dosages. In previous studies, Wang et al. found that live, attenuated CDV vaccines used in giant pandas were inadequate to stimulate enhanced immune responses [15]. A report also suggested that attenuated CDV vaccines for dog had poor efficacy when used in giant panda. The live CDV vaccine can elicit high protective titers for neutralizing antibody (NA) against CDV, but attenuated strains of CDV cannot be safely used in some exotic species, and they may cause symptomatic and sometimes fatal infections in minks and ferrets [16, 17]. Animals receiving attenuated CDV vaccine may become leukocytopenic and develop erythematous rash typical of distemper [18], thereby highlighting the limitations of using this vaccine. And, the safety of CDV vaccine in wild-life also must be proven. More effective and specific immunological preparations should be developed to protect endangered giant pandas, and exploring various vaccine strategies is necessary to produce adequate protective immunity against CDV in giant pandas.

Recently, a nanoparticle-based antigen has received considerable interest because of its multiple advantages over inactivated viruses or subunit soluble antigens. These nanoparticle antigens are obtained from antigenic proteins, which exhibit high immunogenicity [19] and adjuvant effects and stimulate antigen-presenting cells (APCs) upon binding or internalization [20]. Then, they elicit innate and adaptive immunity activation. In our study, we generated nanoparticles from CDV H stabilized with a tetramerization motif to investigate their immunogenicity and role as a potential CDV vaccine in wild animals, such as giant panda.

## Materials and methods

### Ethics statement

This study was approved by the Animal Care and Use Committee of the Jiaxing University with protocol number JUMC2019007. All animal experiments were performed in accordance with the guidelines of the Jiaxing University Animal Care and Use Committee. Immunization and sampling were performed under anesthesia.

### Cell lines and virus gene

Sf9 insect cells were maintained in SF900II (Life Technologies, San Diego, CA, USA) at 27 °C in cell culture, and CDV H gene was obtained from giant panda/SX/2014. A recombinant Onderstepoort strain expressing green fluorescent protein used for virus neutralization was constructed and generated using a reverse genetic system based on RNA polymerase II for CDV by our laboratory.

### Purification and characterization of recombinant CDV hemagglutinin proteins

**Fig. 1:**
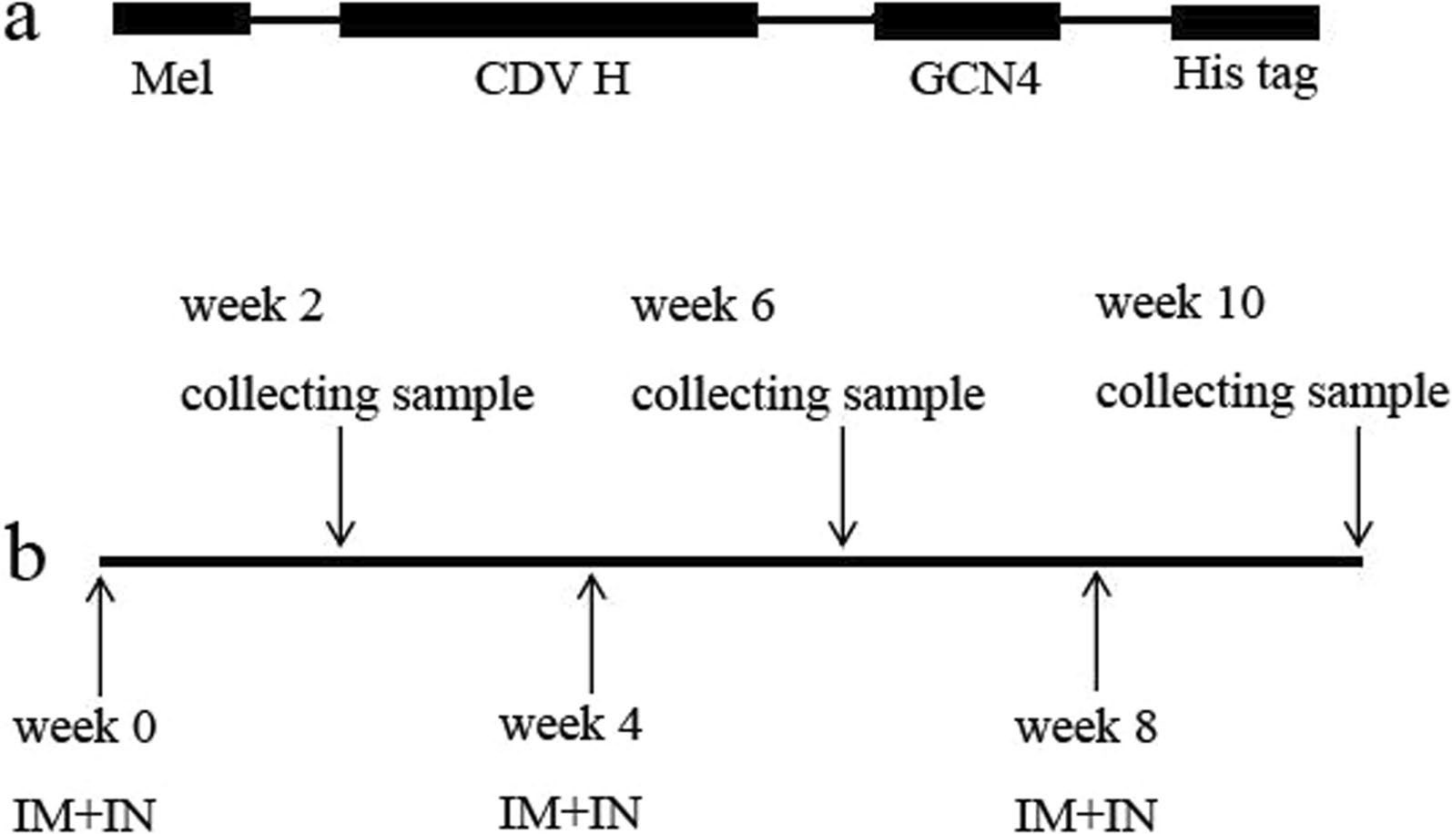
Schematic of constructs and immunization schedule. **a** schematic of constructs expressing full length CDV H containing mellitin SP, CDV H, GCN4, and his tag gene. **b** the mice were immunized three times at 4-week intervals via i.m. and i.n., and samples were collected 2 weeks after each immunization

We constructed recombinant plasmid-GCN4 sequence-stabilized tetrameric H (tH), consisting of a signal peptide-encoding sequence from honeybee melittin to facilitate protein expression in sf9 cells, full-length CDV H gene, foreign tetramerization motif GCN4, and His tag gene at the C-terminal (Fig. 1a). The full CDV H fusion gene was cloned into pFastbacI, and recombinant plasmid was transformed into DH10 Bac *Escherichia coli* (Life Technologies, San Diego, CA, USA) to obtain recombinant bacmid. The bacmid was transfected into sf9 to produce recombinant baculovirus (rBV) after bacmid was identified. rBV expression was generated using a Bac-to-Bac system (Invitrogen, Grand Island, NY, USA). Recombinant H protein was purified by infecting sf9 cells with rBVs at a MOI of 1 and incubated at 27 °C for 48 h. Supernatants were collected, and recombinant CDV H protein was purified using a His tag purification kit (Beyotime, Beijing, China). The sample of infected sf9 cells and pure recombinant CDV H protein was observed by sodium dodecyl sulfate polyacrylamide gel electrophoresis (SDS-PAGE), followed by Western blot analysis. Flagellin expression was determined using the Bac-to-Bac system, purified through his tag, and stored at − 80 ℃ for further use.

### Preparation of nanoparticles

Exactly 1 μg of recombinant CDV H pure protein was incubated at room temperature in the presence of Bis [sulfosuccinimidyl] (BS3) at final concentrations of 6 mM for 30 min. Then, 1 M of Tris–HCl (pH 8.0) was added to reach a final concentration of 50 mM and stop the crosslinking reaction. Then, the sample was separated via SDS-PAGE followed by Western blot analysis using an anti-his tag antibody (Beyotime, Beijing, China) to identify hemagglutinin tetramers (tetrameric hemagglutinin [tH]).

### Immunization and sampling

Female 6-week-old BALB/c mice were randomly divided into three groups of 15 mice each group. The mice were intranasally (i.n.) and intramuscularly (i.m.) immunized with 10 μg of soluble recombinant H protein (group 1, G1), 10 μg of nanoparticle tH (group 2, G2), or 10 μg of nanoparticle tH + 1 μg of flagellin (group 3, G3) at weeks 0, 4, and 8, respectively (Fig. 1b). Based on our previous reports, flagellin is an effective mucosal adjuvants [19]. Sera and nasal samples were collected 2 weeks after each immunization (we tested the samples collected previously). Lymphocytes from the spleen and lymph node were collected after the last sample was collected and used for ELISPOT testing. Inguinal lymph nodes were collected after primary immunization for flow cytometry assays.

### Neutralization assay and ELISA

CDV H-specific antibody titers, IgG, IgG1, IgG2a, and IgA in immune samples were detected by ELISA using purified recombinant CDV H protein as coating antigens at 1 μg/mL. The diluted samples were added to each well and incubated. After washing, the plates were incubated with HRP-conjugated goat anti-mouse IgG, IgG1, IgG2a, and IgA antibodies (Southern Biotechnology Associates, Birmingham, AL, USA). TMB was used to develop the color, and an ELISA reader was used to read the OD value at 450 nm.

Neutralizing antibody was assessed by incubating the double dilution of serum with 100 TCID_50_ CDV for 1 h and adding it into vero cells at 10^5^ cells/well in 96-well plates. The NA titers were calculated using the method of Reed and Muench.

### Cytokine ELISpot

Interferon gamma (INF-γ) and interleukin 4 (IL-4) secretions from immunized mouse splenocytes and lymph node cells were evaluated using ELISpot kits (eBioscience, San Diego, CA) in accordance with the manufacturer’s instructions.

### Flow cytometry assays for DCs

The lymph nodes were collected at 3, 6, and 9 days after primary immunization. Single-cell suspensions (1 × 10^6^ cells/mL) were prepared in PBS containing 2% FBS and stained with anti-mouse CD11c, CD80, and CD86 antibodies (BD Biosciences, Franklin, TN, USA) for 30 min at 4 °C. After staining, the labeled cells were washed two times with PBS containing 2% FBS and analyzed using a flow cytometer.

### Statistical analysis

The analyses were performed by using GraphPad Prism version 5.00 for Windows (GraphPad Software, San Diego, CA). *P*-values less than 0.05 (*P < 0.05) were considered to be statistically significant: **P < 0.01; ***P < 0.001; n.s., *P* > 0.05.

## Results

### Characterization of CDV H and nanoclusters

**Fig. 2:**
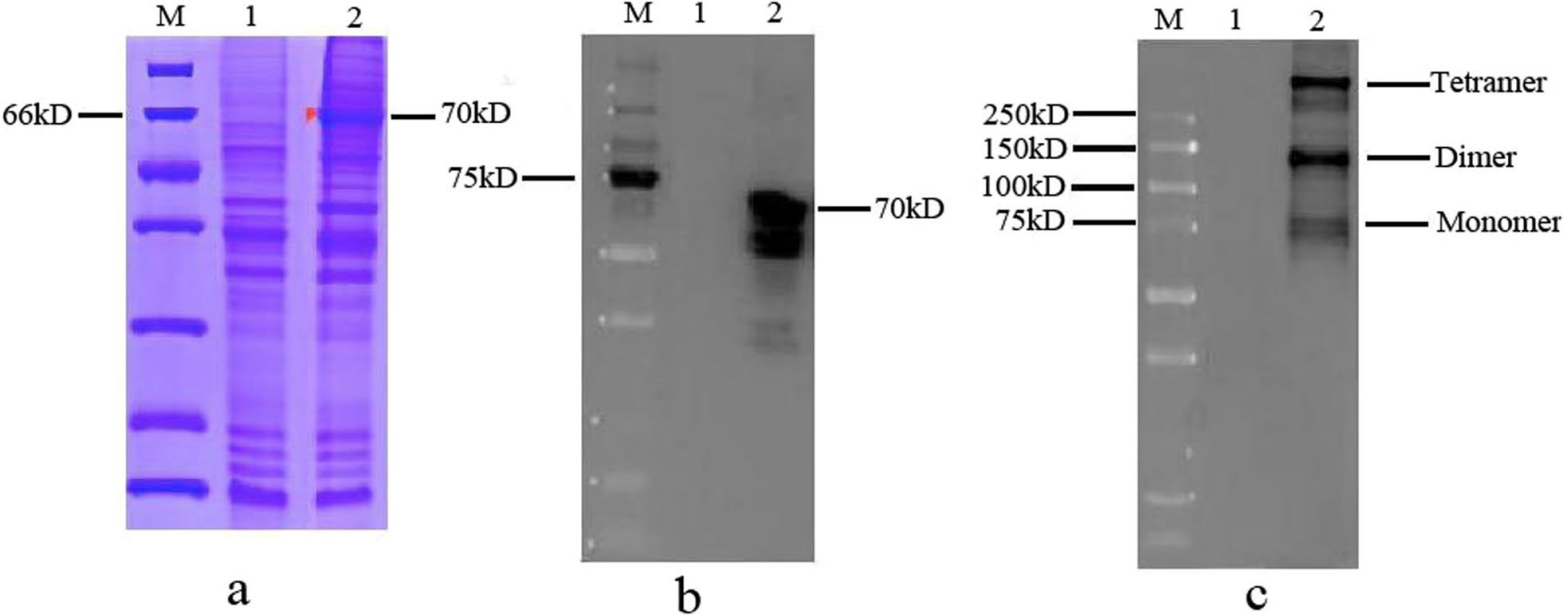
Generation and characterization of CDV H protein and nanoparticle tH. **a** and **b** Coomassie blue staining and Western blot analysis of CDV H protein expression, M, molecular weight (kD), lane 1, sf9 cells, lane 2, sf9 cells infected with recombinant baculovirus; **c**, CDV H protein was purified, and cross-linked CDV H samples were applied to Western blot analysis, M, molecular weight (kD), lane 1, sf9 cells, lane 2, cross-linked CDV H protein

Figure 2a shows that the CDV H protein was expressed in sf9 cells. Lane 1 shows the sf9 cell sample, whereas lane 2 shows the H protein in sf9 cells. The CDV recombinant H protein has a molecular mass of approximately 70 kDa. Figure 2b shows the Western blot results for purified recombinant H protein. The tetrameric structure of tH was confirmed by using BS3 for fixing, followed by cross-linking reaction. Western blot analysis showed a major band with a molecular mass of 280 kDa, representing the CDV H tetramer, and a band with a molecular mass of 140 kDa, representing the dimers. A band with a molecular mass of 70 kDa represented the CDV H monomer (Fig. 2c). Thus, GCN4-stabilized recombinant H had a tetrameric form.

### Tetrameric CDV H nanoparticles induced strong humoral responses

**Fig. 3:**
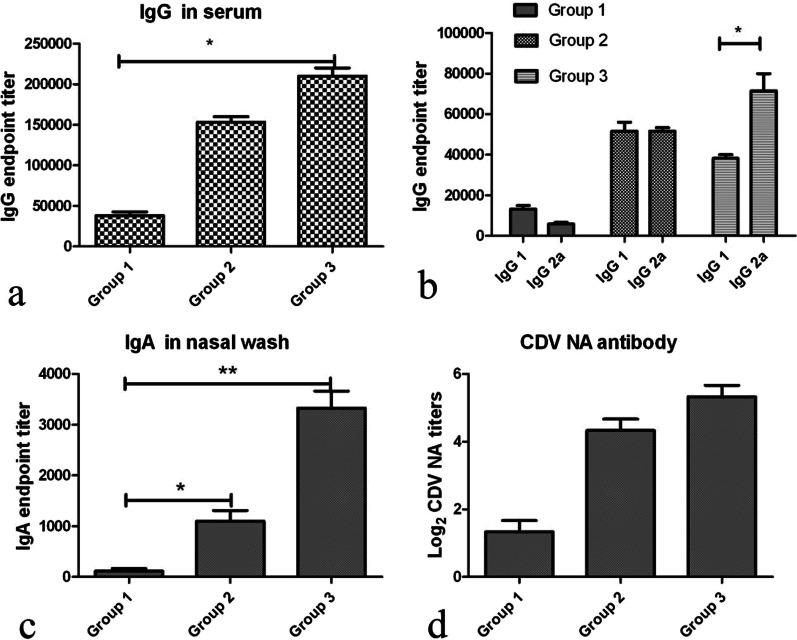
Systemic and mucosal antibody responses against CDV. The mice were immunized with soluble CDV H and nanoparticles, and sera and nasal wash samples were collected 2 weeks after each immunization. The final samples of tested data are shown. ELISA plates were coated with soluble CDV H protein, and the samples were diluted 2 × stepwise. Bound antibody was detected by binding HRP-conjugated goat anti-mouse IgG, IgG1, IgG2a, and IgA. A, serum IgG titers; B, IgG isotypes; C, IgA titers of nasal wash; D, CDV NA titers. Assays were performed as described in materials and methods. Results are expressed as means ± standard deviations. *P* < 0.05 was considered statistically significant.**P < 0.01, * *P* < 0.05

The efficiency of tH as an immunogen was examined by immunizing mice with soluble CDV H protein and nanoparticle tH with or without flagellin. Then, immune responses, including systemic and mucosal immune responses, were tested. Serum and mucosal samples were evaluated for CDV H-specific IgG and IgA titers by using ELISA. As shown in Fig. 3a, c, nanoparticle tH with or without flagellin elicited significantly higher IgG and IgA titers compared with the soluble CDV H group. Mice immunized with nanoparticles achieved between sevenfold and eightfold higher IgG titers than mice from G1. The mice from G3 (nanoparticles + flagellin) also showed enhanced immune responses compared with those from G2, but this difference was not significant.

We also compared the IgG isotype. As shown in Fig. 3b, in G3, the high levels of antibody, primarily the IgG2a isotype (IgG1/IgG2a around 0.5; *P* < 0.05), were induced. In G1, the predominantly dominant IgG1 humoral antibody responses were induced (IgG1/IgG2a around 2; *P* < 0.05). In G2, tH induced Th1 and Th2 immune responses (IgG1/IgG2a around 0.93; *P* > 0.05).

When sera were examined for virus neutralization, only the mice immunized with tH, in G2 and G3, had increased NA titers (Fig. 3d), and only the background levels of NA were detected in G1.

### Tetrameric CDV H nanoparticles induce robust mucosal antibody responses

We tested CDV H-specific IgA antibody in nasal wash by ELISA to evaluate whether immunization with nanoparticles can elicit mucosal antibody responses. As shown in Fig. 3c, in G2 and G3, significantly higher levels of IgA were observed, and nanoparticles dramatically improved IgA immuno responses compared with G1. Mice immunized with nanoparticles achieved more than tenfold higher IgA titers than those from G1, and IgA antibody responses in G2 were significantly increased compared with those in G1.

### CDV H nanoparticle activation of DCs in lymph nodes

**Fig. 4:**
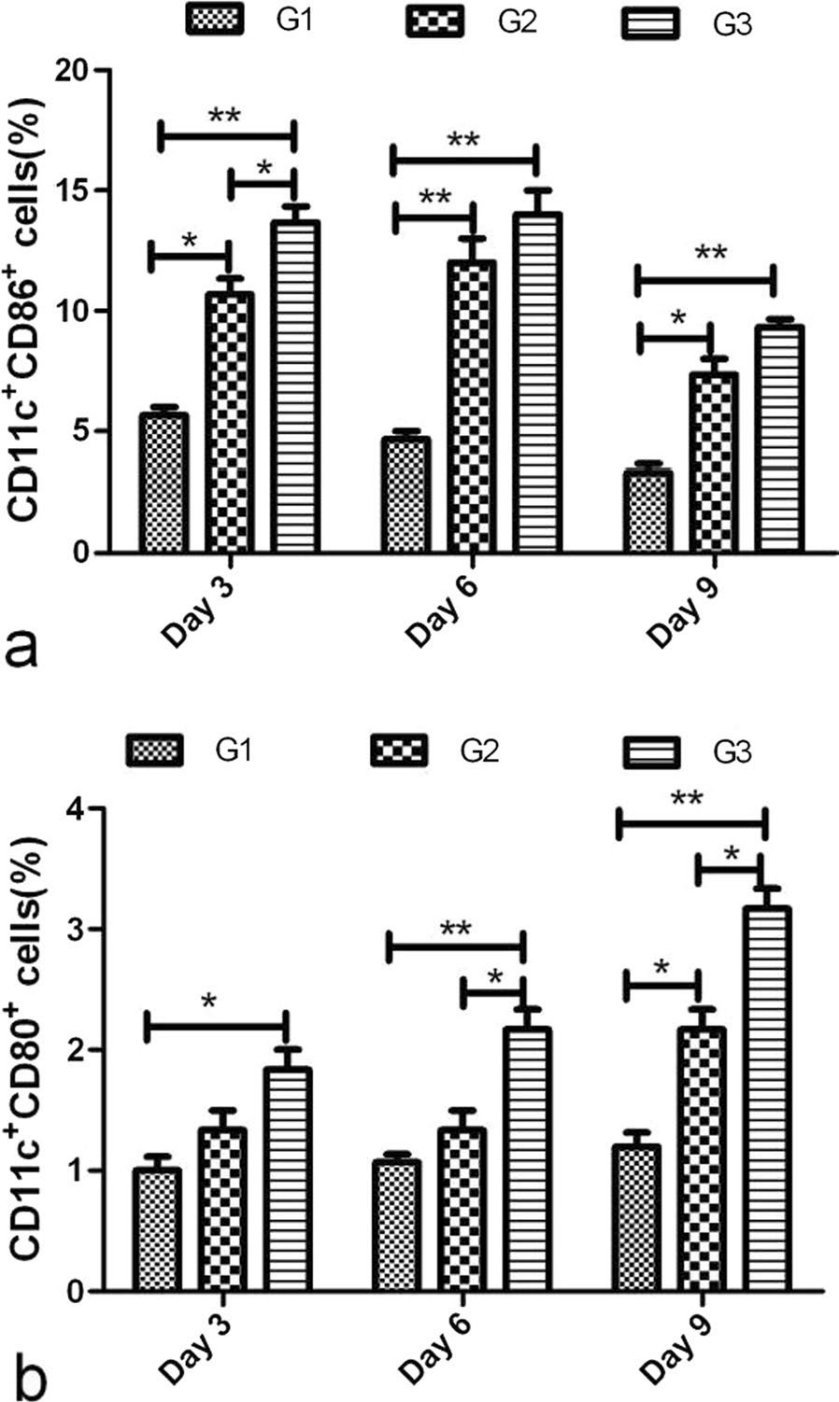
Flow cytometry assay of DCs in lymph nodes. The lymph nodes were collected from each group 3, 6, and 9 days after the first immunization. The cells were stained with mouse anti-CD11c, -CD86, and -CD80 monoclonal antibodies. Double positive cells that CD11c^+^CD86^+^ (**a**); CD11c^+^CD80^+^ (**b**) were plotted, and the data represent the means of double positive cells percentage, * *P* < 0.05, ** *P* < 0.01

Lymph node cells were analyzed by flow cytometry to investigate whether nanoparticles can stimulate DC activation. Figure 4 shows that more DCs (CD11c + CD86 + and CD11c + CD80 + cells) were detected in mice from G2 and G3 than those in G1. CD86 and CD80 expression in G2 and G3 was higher compared with that in G1, and this difference was statistically significant. These results indicated that nanoparticles elicited a higher percentage of co-stimulatory expression of DCs compared with the soluble CDV H protein-immunized group.

### Tetrameric CDV H nanoparticles activate CDV H-specific T cell responses

**Fig. 5:**
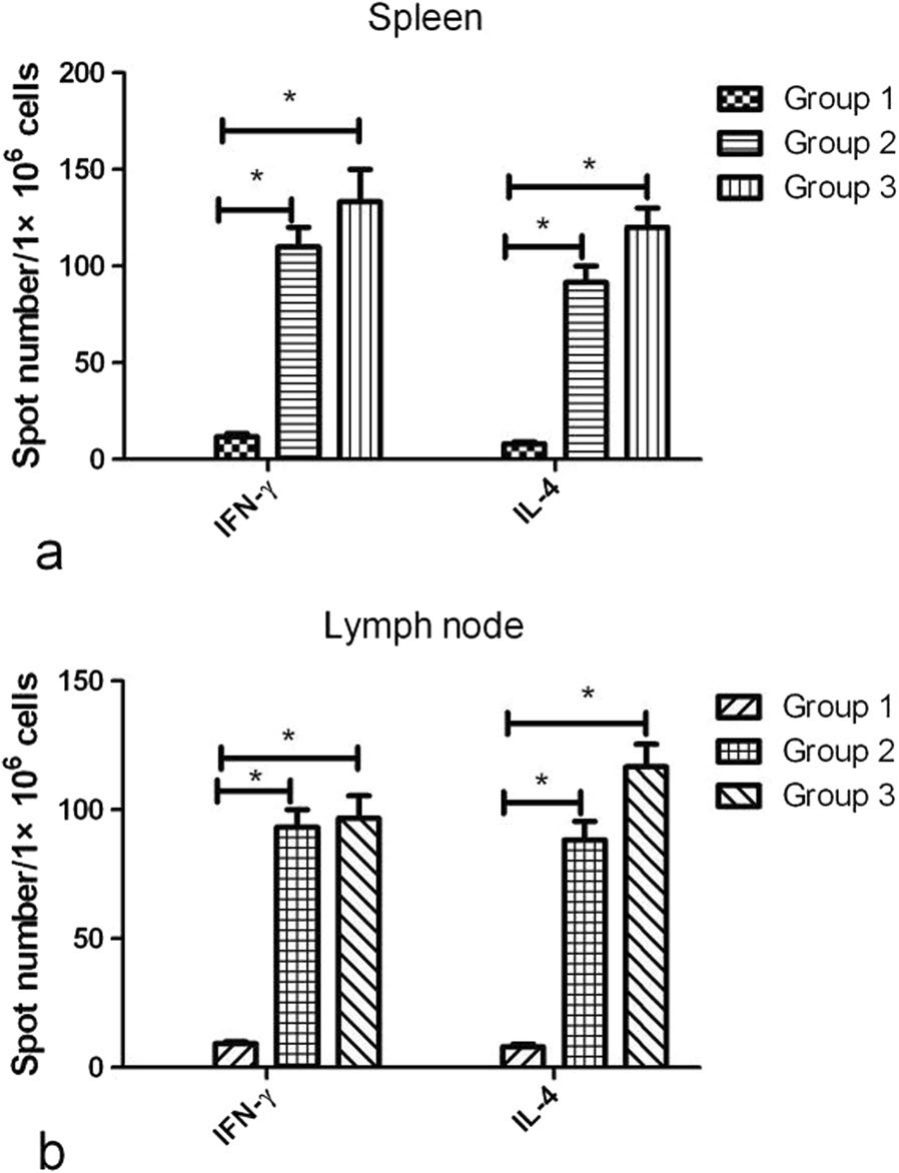
ELISpot analysis of CDV H-specific IFN-γ and IL-4 secretion. The spleen and lymph node were collected from each group 2 weeks after the final immunization. Lymphocytes from the spleen and lymph node were stimulated with soluble CDV H protein, tH, and flagellin. The IFN-γ and IL-4 cytokine-secreting cells were determined using ELISpot assay. The secretion of IFN-γ and IL-4 in the spleen (**a**) and lymph node (**b**). * *P* < 0.05, **P < 0.01

T-cell responses are important and known to contribute to broad cross protection. IFN-γ- and IL-4-secreting cells in the spleens and lymph node of immunized mice were evaluated using cytokine ELISpot. As shown in Fig. 5, in G2 and G3, the IFN-γ-secreting cells in the spleens and lymph node were significantly higher after stimulation with tH compared with mice immunized with recombinant soluble CDV H protein. The IL-4-secreting cells were also detected in the spleens and lymph node of mice in G2 and G3 compared with those immunized with recombinant soluble CDV H protein, and this difference was statistically significant. Only background levels of cytokine-secreting cells were detected in G1, and these results showed that nanoparticles induced enhanced CDV H-specific T-cell responses compared with G1.

## Discussion

Many studies have shown that protein in particle form can enhance immunogenicity because it mimics the natural conformation of this protein in viral particles, and these particles provide a suitable route of vaccine administration and enhance cellular uptake, thereby resulting in robust, innate, humoral, cellular, and mucosal immune responses, conferring complete protection against lethal virus challenge [21, 22]. Given the limitations of the current CDV vaccine, we generated a terminal tetramerization module based on the leucine-zipper domain, which spontaneously assembled into a parallel four-helix bundle-tetrameric CDV H nanoparticles, and tested its immunogenicity.

In the present study, we examined the effects of CDV H nanoparticles, and high IgG titers were observed in the three groups, particularly in G2 and G3. Nanoparticle antigens elicited efficient immune response, which may involve antigen density and distribution on the pathogen. T and B cell activation relies on effective cross-linking between B cell receptor (BCR) and the recognition pattern presented by the pathogen. The high-density ordered antigenic array provides multiple binding events, which occur simultaneously between the nanoparticle antigen and BCR, and promotes cellular activation effectively, compared with the effect of monovalent binding caused by single soluble recombinant antigens [23]. The IgG isotype profiles may reflect the Th cells that are activated in the early stage of immunization or infection and suggest the suitable mechanism of antibody-mediated effector functions to be used [24]. In our study, G1 indicated a Th2 response, whereas the inoculation of nanoparticles in G2 induced a Th1/Th2 balance response. Th1-based responses with an IgG2a-dominant IgG isotype was observed in G3. Th1-based response confers anti-viral effect by secreting antiviral molecules and stimulating cellular effect to kill infected cells, whereas Th2-based response confers effect by helping B cell differentiation and proliferation to produce better antibody response [24].

When mice were vaccinated with nanoparticles, they developed CDV-NA titer comparable to the soluble-protein-immunized group. Neutralizing antibodies are critical factors for the elimination of free viral particles and clinical prognosis of infected animals. Neutralizing antibodies are responsible for preventing intra and extracellular viral dissemination. In G1, only background NA response was tested. Nanoparticle tH elicited high NA titer in G2 and G3, but the difference was not statistically significant compared with that in G1. Brindley et al. engineered headless MeV-H stem constructs capable of efficiently inducing MeV F refolding; combined with previous work, the findings indicate that the introduction of terminal tetramerization tag to the MeV H stalk can induce covalent H tetramerization [25, 26]. This result further confirms that the four-helix bundle structure represents the conserved physiological configuration of paramyxovirus attachment protein stalk [27]. Maintaining or mimicking the native conformation of CDV H may explain the mechanism through which nanoparticle tH induced high levels of spatial conformation-dependent NA titer in our study.

CDV is transmitted by aerosols, which infects the upper respiratory tract, and replicated in the macrophages and lymphocyte, and viral particles spread to bronchial lymph nodes and tonsils; the viral replication in lymphoid tissues lead to lasting and severe immunosuppression [28, 29]. Thus, mucosal immune responses are important for CDV immune protection; IgA in upper respiratory tract secretions plays a major role in antiviral immunity [30] and contributes to protective immunity. We found that the IgA levels in G2 and G3 were enhanced. The IgA antibody titer in G2 and G3 was significantly different compared with that in G1. Flagellin is an effective adjuvant used in many studies, which induces strong systemic and mucosal immune responses. Our study also found that mixed flagellin elicited high titers of IgG and IgA.

DCs play an important role in stimulating the proliferation and differentiation of naive and memory T cells. DC activation in the lymph node in G2 and G3 was remarkable. The co-stimulatory CD80 and CD86 expression was upregulated, which is often referred to as the second signal and is essential for the induction of effector T cells. The size of nanoparticle antigens is favorable for DC sample. DCs process and present the antigens to T-cells to activate cellular immune responses and then modulate the functions of B cells. The activation of DCs plays an important role in adaptive immune responses. DCs are the targets of flagellin in initiating the TLR5-associated innate signaling pathway [31]. In G3, nanoparticles with flagellin may efficiently utilize the innate-signaling function of flagellin, and the activation of these APCs promotes antigen presentation and cytokine production, which drives antigen-specific adaptive responses. In addition to flagellin activation, nanoparticles that are highly symmetrical, stable, and structurally organized mimic the repetitive surface architecture of a natural microbe, and nanoparticles with diameters of 100–200 nm are highly suitable for optimal interactions with various cells of the immune system [32]. These in vivo data indicate that nanoparticles can induce high levels of cytokine secretion and enhance the co-stimulatory molecular expression on the surface of DCs, thereby stimulating DC maturation. In brief, nanoparticles can enhance antigen adsorption and uptake by APCs, facilitate antigen processing, induce maturation of DCs, promote antigen cross-presentation, and induce the production of innate cytokines that regulate humoral and cellular immune responses.

The host defense depends on the innate immune system, which is also responsible for producing signals that activate the adaptive immunity. The IFN are critical elements in the innate immune defense against viruses. Cytokine analysis shows that G2 and G3 produce more IFN-γ and IL-4-secreting T cells, particularly in G3, demonstrating that nanoparticles enhance T cell responses in mice. Cellular immune response is critical in viral clearance, and a vigorous and continued cellular immunity, including cytotoxic T and killer cells, can determine CDV elimination in infected animals [33].

## Conclusions

In this work, high IgG and IgA antibody titers were detected in nanoparticle-immunized mice. Moreover, the nanoparticle-immunized group showed stronger T cell response and NA compared with the other group. Collectively, the results showed that the vaccine could induce increased immunogenicity and strong immune response; thus, it is a potential novel vaccine for CDV in wild-life. In addition, MV and CDV belong to the genus *Morbillivirus*, which have similar pathogenesis, and they enter the hosts through the respiratory tract and target immune cells residing within the airways. Our results provide the suitable vaccine for MV.

## Data Availability

The datasets used and/or analysed during the current study are available from the corresponding author on reasonable request.

## Abbreviations

CDV: Canine distemper virus
MV: Measles virus
APC: Antigen-presenting cells
VN: Virus neutralizing
NA: Neutralizing antibody
tH: Tetrameric hemagglutinin
CD: Canine distemper
INF-γ: Interferon gamma
IL-4: Interleukin 4
